# Tobacco Smoking and Mortality in Asia: A Pooled Meta-analysis

**DOI:** 10.1001/jamanetworkopen.2019.1474

**Published:** 2019-03-29

**Authors:** Jae Jeong Yang, Danxia Yu, Wanqing Wen, Xiao-Ou Shu, Eiko Saito, Shafiur Rahman, Prakash C. Gupta, Jiang He, Shoichiro Tsugane, Yong-Bing Xiang, Yu-Tang Gao, Woon-Puay Koh, Akiko Tamakoshi, Fujiko Irie, Atsuko Sadakane, Ichiro Tsuji, Seiki Kanemura, Keitaro Matsuo, Chisato Nagata, Chien-Jen Chen, Jian-Min Yuan, Myung-Hee Shin, Sue K. Park, Wen-Harn Pan, You-Lin Qiao, Mangesh S. Pednekar, Dongfeng Gu, Norie Sawada, Hong-Lan Li, Jing Gao, Hui Cai, Eric Grant, Yasutake Tomata, Yumi Sugawara, Hidemi Ito, Keiko Wada, Chen-Yang Shen, Renwei Wang, Yoon-Ok Ahn, San-Lin You, Keun-Young Yoo, Habibul Ashan, Kee Seng Chia, Paolo Boffetta, Manami Inoue, Daehee Kang, John D. Potter, Wei Zheng

**Affiliations:** 1 Division of Epidemiology, Department of Medicine, Vanderbilt Epidemiology Center, Vanderbilt-Ingram Cancer Center, Vanderbilt University Medical Center, Nashville, Tennessee; 2 Division of Cancer Statistics Integration, Center for Cancer Control and Information Services, National Cancer Center, Tokyo, Japan; 3 Department of Global Health Policy, Graduate School of Medicine, University of Tokyo, Tokyo, Japan; 4 Healis-Sekhsaria Institute for Public Health, Mahape, Navi Mumbai, India; 5 Department of Epidemiology, Tulane University School of Public Health and Tropical Medicine, New Orleans, Louisiana; 6 Epidemiology and Prevention Group, Center for Public Health Sciences, National Cancer Center, Tokyo, Japan; 7 State Key Laboratory of Oncogene and Related Genes, Department of Epidemiology, Shanghai Cancer Institute, Renji Hospital, Shanghai Jiaotong University School of Medicine, Shanghai, People’s Republic of China; 8 Duke-NUS Medical School Singapore, Singapore, Republic of Singapore; 9 Saw Swee Hock School of Public Health, National University of Singapore, Singapore, Republic of Singapore; 10 Graduate School of Medicine, Hokkaido University, Sapporo, Japan; 11 Ibaraki Chikusei Public Health Center, Chikusei City, Japan; 12 Radiation Effects Research Foundation, Hiroshima, Japan; 13 Tohoku University Graduate School of Medicine, Sendai, Japan; 14 Division of Molecular and Clinical Epidemiology, Aichi Cancer Center Research Institute, Nagoya, Japan; 15 Department of Epidemiology, Nagoya University Graduate School of Medicine, Nagoya, Japan; 16 Graduate School of Medicine, Gifu University, Gifu City, Japan; 17 Academia Sinica, Taipei City, Taiwan; 18 Graduate School of Public Health, University of Pittsburgh, Pittsburgh, Pennsylvania; 19 Department of Social and Preventive Medicine, Sungkyunkwan University School of Medicine, Seoul, South Korea; 20 Department of Preventive Medicine, Seoul National University College of Medicine, Seoul, South Korea; 21 Department of Biomedical Sciences, Seoul National University Graduate School, Seoul, South Korea; 22 Cancer Research Institute, Seoul National University, Seoul, South Korea; 23 Institute of Biomedical Sciences, Academia Sinica, Taipei City, Taiwan; 24 Cancer Foundation of China, Beijing, People’s Republic of China; 25 Fuwai Hospital, National Center for Cardiovascular Diseases, Chinese Academy of Medical Sciences and Peking Union Medical College, Beijing, People’s Republic of China; 26 Taiwan Biobank, Institute of Biomedical Sciences, Academia Sinica, Taipei City, Taiwan; 27 Graduate Institute of Environmental Science, China Medical University, Taichung, Taiwan; 28 School of Medicine, Big Data Research Center, Fu Jen Catholic University, Taipei City, Taiwan; 29 Armed Forces Capital Hospital, Seoul National University College of Medicine, Seoul, South Korea; 30 Cancer Research Center, Department of Health Studies, University of Chicago, Chicago, Illinois; 31 Cancer Research Center, Department of Medicine, University of Chicago, Chicago, Illinois; 32 Cancer Research Center, Department of Human Genetics, University of Chicago, Chicago, Illinois; 33 Icahn School of Medicine at Mount Sinai, New York, New York; 34 Division of Public Health Sciences, Fred Hutchinson Cancer Research Center, Seattle, Washington; 35 Centre for Public Health Research, Massey University, Wellington, New Zealand; 36 Department of Epidemiology, University of Washington, Seattle

## Abstract

**Importance:**

Understanding birth cohort–specific tobacco smoking patterns and their association with total and cause-specific mortality is important for projecting future deaths due to tobacco smoking across Asian populations.

**Objectives:**

To assess secular trends of tobacco smoking by countries or regions and birth cohorts and evaluate the consequent mortality in Asian populations.

**Design, Setting, and Participants:**

This pooled meta-analysis was based on individual participant data from 20 prospective cohort studies participating in the Asia Cohort Consortium. Between September 1, 2017, and March 31, 2018, a total of 1 002 258 Asian individuals 35 years or older were analyzed using Cox proportional hazards regression analysis and random-effects meta-analysis. The pooled results were presented for mainland China; Japan; Korea, Singapore, and Taiwan; and India.

**Exposures:**

Tobacco use status, age at starting smoking, number of cigarettes smoked per day, and age at quitting smoking.

**Main Outcomes and Measures:**

Country or region and birth cohort–specific mortality and the population attributable risk for deaths from all causes and from lung cancer.

**Results:**

Of 1 002 258 participants (51.1% women and 48.9% men; mean [SD] age at baseline, 54.6 [10.4] years), 144 366 deaths (9158 deaths from lung cancer) were ascertained during a mean (SD) follow-up of 11.7 (5.3) years. Smoking prevalence for men steadily increased in China and India, whereas it plateaued in Japan and Korea, Singapore, and Taiwan. Among Asian male smokers, the mean age at starting smoking decreased in successive birth cohorts, while the mean number of cigarettes smoked per day increased. These changes were associated with an increasing relative risk of death in association with current smoking in successive birth cohorts of pre-1920, 1920s, and 1930 or later, with hazard ratios for all-cause mortality of 1.26 (95% CI, 1.17-1.37) for the pre-1920 birth cohort, 1.47 (95% CI, 1.35-1.61) for the 1920s birth cohort, and 1.70 (95% CI, 1.57-1.84) for the cohort born in 1930 or later. The hazard ratios for lung cancer mortality were 3.38 (95% CI, 2.25-5.07) for the pre-1920 birth cohort, 4.74 (95% CI, 3.56-6.32) for the 1920s birth cohort, and 4.80 (95% CI, 3.71-6.19) for the cohort born in 1930 or later. Tobacco smoking accounted for 12.5% (95% CI, 8.4%-16.3%) of all-cause mortality in the pre-1920 birth cohort, 21.1% (95% CI, 17.3%-24.9%) of all-cause mortality in the 1920s birth cohort, and 29.3% (95% CI, 26.0%-32.3%) of all-cause mortality for the cohort born in 1930 or later. Tobacco smoking among men accounted for 56.6% (95% CI, 44.7%-66.3%) of lung cancer mortality in the pre-1920 birth cohort, 66.6% (95% CI, 58.3%-73.5%) of lung cancer mortality in the 1920s birth cohort, and 68.4% (95% CI, 61.3%-74.4%) of lung cancer mortality for the cohort born in 1930 or later. For women, tobacco smoking patterns and lung cancer mortality varied substantially by countries and regions.

**Conclusions and Relevance:**

In this study, mortality associated with tobacco smoking continued to increase among Asian men in recent birth cohorts, indicating that tobacco smoking will remain a major public health problem in most Asian countries in the coming decades. Implementing comprehensive tobacco-control programs is warranted to end the tobacco epidemic.

## Key Points

**Question:**

How are secular trends of tobacco smoking associated with mortality across countries or regions by birth cohorts in Asia?

**Findings:**

This pooled meta-analysis of 1 002 258 participants in 20 prospective cohort studies shows that smoking-attributable mortality continued to increase among Asian men in successive birth cohorts, with the tobacco smoking epidemic worsening in the past few decades.

**Meaning:**

Tobacco smoking will remain a significant public health problem in most Asian countries, stressing the need to implement and enhance comprehensive tobacco-control programs in Asia.

## Teaser

This pooled meta-analysis of more than 1 million participants assesses secular trends of tobacco smoking by countries or regions and birth cohorts and evaluates the association with mortality in Asian populations.

## Introduction

Globally, more than 7 million people die each year as a result of tobacco smoking.^1^ The number of deaths attributable to smoking is projected to increase to 8.3 million by 2030, with the largest increase in low- and middle-income countries.^2^ If current smoking patterns persist, it is estimated that 1 billion deaths will occur in this century as a result of tobacco use.^3^ The overall mortality and morbidity attributed to tobacco smoking have declined steadily during the past few decades in many Western countries; however, in Asia, a tobacco epidemic has developed rapidly.^4,5,6^ Currently, about half the world’s male smokers live in 3 Asian countries: China, India, and Indonesia.^7^ Japan and Bangladesh also rank among the top 10 countries with the largest smoking populations.^3,7^ Not only is Asia the world’s largest tobacco consumer, it is also the largest tobacco producer.^3,4,8^

Asian countries are in the early stages of the tobacco smoking epidemic.^4,9,10^ During the last century, many Asian countries experienced dynamic socioeconomic changes, such as rapid economic development, westernization, and war. The timing of these events may have induced different patterns of tobacco use across birth cohorts (the group of people born in given calendar years). Studies conducted in Japan and China have found a distinct birth cohort–specific smoking pattern that has resulted in a different risk of death associated with tobacco smoking.^11,12,13,14^ Understanding patterns of tobacco use across birth cohorts and countries will provide valuable data to determine the future burden of smoking-attributable mortality in Asia. Currently, little is known about the evolution of the tobacco smoking epidemic and its consequences on mortality by birth cohorts across Asian populations.

In this study, we pooled individual-level data from more than 1 million participants in 20 Asian cohort studies and assessed the secular trends of tobacco smoking by countries or regions and birth cohorts. We quantified the country-specific or region-specific and birth cohort–specific risks of death from all causes and from lung cancer associated with tobacco smoking. Based on these risk estimates, we further evaluated the burden of mortality attributable to tobacco smoking by birth cohorts in an attempt to identify trends in tobacco use and the consequent mortality in Asian populations.

## Methods

### Study Populations

This pooled meta-analysis is based on individual participant data from 20 prospective cohort studies in the Asia Cohort Consortium,^4,15^ including cohorts from studies conducted in mainland China, Japan, South Korea, Singapore, Taiwan, and India (eFigure 1 in the Supplement). Specific descriptions on participating cohorts are given in eTable 1 in the Supplement. Briefly, each cohort conducted a baseline survey to collect information on sociodemographic characteristics, lifestyle factors (including tobacco smoking), and medical history from study participants who were subsequently followed up to monitor their health outcomes. Deidentified data from study participants were harmonized at the Asia Cohort Consortium coordinating center (further details are given in the eAppendix in the Supplement). All participating cohorts were approved by their research ethics committees. Our study follows the Strengthening the Reporting of Observational Studies in Epidemiology (STROBE) reporting guideline.

Of the 1 113 112 individuals included in these cohorts, 27 882 were excluded because of invalid data on smoking status, birth year, vital status, or follow-up periods. To minimize the potential influence of reverse causation, we excluded 76 023 individuals who had a history of cancer or cardiovascular disease at baseline or who died or were lost to follow-up within the first year after enrollment. Given that tobacco-associated deaths are uncommon in early adulthood, another 6949 individuals who were younger than 35 years were excluded. After these exclusions, 1 002 258 participants remained as the final analytic sample.

### Smoking Assessment

Information on self-reported tobacco smoking was obtained at the baseline survey. Data on bidi use, which is a type of hand-rolled cigarette filled with tobacco flakes, was additionally assessed for the Mumbai cohort given its popularity in South Asia. Ever smokers were defined slightly differently across studies but were typically defined as those who had smoked at least 1 cigarette per day for at least 6 months or had smoked at least 20 packs of cigarettes in their life. Smoking status was defined using data collected at baseline as lifelong never smokers, former smokers, and current smokers. For ever smokers, information on age at which the participant started and/or quit smoking and the number of cigarettes smoked per day was collected.

### Outcomes

Death outcomes were ascertained via data linkages to death certificates and/or active follow-up surveys. The cause of death was defined using the *International Classification of Diseases, Ninth Revision* (*ICD-9*) or *International Statistical Classification of Diseases and Related Health Problems, Tenth Revision* (*ICD-10*). The primary outcomes of this study were deaths from all causes or from lung cancer (*ICD-9* code, 162 and *ICD-10* code, C34). Study participants were censored at the date of death, end of follow-up, or loss to follow-up, whichever came first.

### Statistical Analysis

To evaluate birth cohort patterns of smoking behaviors, birth cohorts were classified into pre-1910, 1910s, 1920s, 1930s, 1940s, and 1950 or later. Prevalence of current and ever smoking, mean age at starting smoking, and mean number of cigarettes smoked per day across birth cohorts were illustrated by sex and country or region. Mean age at quitting smoking was compared to assess the cessation pattern by baseline age (<50, 50-59, 60-69, and ≥70 years) and birth cohort.

The birth cohort–specific relative risks of death were quantified via a 2-stage meta-analysis method.^16^ First, Cox proportional hazards regression was used to estimate hazard ratios (HRs) and 95% CIs of death associated with tobacco smoking by birth cohorts (collapsed to <1920, 1920s, and ≥1930 for the stability of point estimates) in each study. Then, study-specific risk estimates were pooled with random-effects meta-analysis (eAppendix in the Supplement).^17^ Age at enrollment (entry) and age at censoring (exit) were treated as the time scale. To consider the period effect across study populations, models were stratified into 5-year groups by birth year and enrollment year. Using never smokers as the reference, we estimated relative risks for all-cause mortality and lung cancer mortality linked to current and ever smoking, age at starting smoking (<20, 20-24, 25-29, and ≥30 years), number of cigarettes smoked per day (<10, 10-19, 20-29, and ≥30; 4 bidis were converted to 1 cigarette^4^), and age at quitting smoking (<40, 40-49, 50-59, and ≥60 years). Covariates included baseline age, body mass index (calculated as weight in kilograms divided by height in meters squared: <18.5, 18.5-24.99, 25.0-29.99, and ≥30.0), urban or rural residence, educational level (no schooling, primary education, secondary education, trade or technical school, university graduate, and graduate studies), and marital status (single, married, and other). These variables were treated as fixed covariates in the models. Missing covariates were assigned the median (continuous) or mode (categorical) values of cohort-specific nonmissing covariates. The proportional hazards assumption for all variables was checked using Schoenfeld residuals, and no violation was found. All analyses were performed separately by sex given the differences in both smoking and mortality patterns between men and women. Country-specific or region-specific results are presented for China; Japan; Korea, Singapore, and Taiwan; and India. Because of the small sample size, South Korea, Singapore, and Taiwan were combined based on their similar stages of economic development. Also, given the differences in smoking patterns and economic development status between rural and urban China,^11^ analyses were conducted separately for each area; the results for rural China are shown in eFigure 2 in the Supplement due to the small sample size.

The population attributable risk (PAR) and the corresponding 95% CI for deaths from all causes and lung cancer were calculated with the following equation^18^: *PAR* = [*P* × (*RR* − 1)]/{[*P* × (*RR* − 1)] + 1}, where *P* is smoking prevalence and *RR* is relative risk (adjusted HRs in the present analyses) for ever smokers compared with never smokers. By using pooled risk estimates, PARs were calculated for each country or region and for the total population. Between September 1, 2017, and March 31, 2018, all analyses were conducted using SAS, version 9.3 (SAS Institute Inc).

## Results

Of 1 002 258 participants, we ascertained 144 366 deaths (9158 deaths from lung cancer) during a mean (SD) follow-up of 11.7 (5.3) years. The prevalence of ever smoking was 65.4% for men and 7.8% for women. The mean (SD) age at which participants started smoking was 22.8 (7.4) years (22.1 [6.6] years for men and 28.2 [10.9] years for women), and the mean (SD) number of cigarettes smoked per day was 16.5 (11.5) (17.2 [11.7] cigarettes for men and 11.2 [8.3] cigarettes for women; Table 1). Baseline characteristics across birth cohorts are presented in eTable 2 in the Supplement.

**Table 1.** Characteristics of Participating Cohorts in the Asia Cohort Consortium

Participating Cohort	Participants, No.^a^	Baseline Survey Years	Follow-up Period, y	Women, %	Mean Age at Baseline, y^b^	Ever Smokers, %		Mean Age at Starting Smoking, y^c^	Mean Cigarettes Smoked/d, No.^d^	Deaths, No.	
						Men	Women			All Causes	Lung Cancer
China											
CHEFS	137 569	1990-1992	7.8	50.9	55.4	63.9	13.4	22.9	13.2	14 885	880
SCS	18 010	1986-1989	16.4	0	55.2	57.2	NA	25.2	16.1	4902	621
SMHS	56 165	2002-2006	9.6	0	54.4	70.4	NA	23.2	16.5	3886	573
SWHS	67 268	1997-2000	15.0	100	51.8	NA	2.7	35.0	9.4	5637	515
Japan											
3Pref Aichi	29 691	1985-1985	11.9	50.5	56.1	84.3	17.5	22.3	21.2	5343	352
IPHS	92 076	1993-1994	11.6	66.4	58.4	77.8	5.6	NA	NA	9560	NA
JACC	74 632	1988-1990	12.9	56.4	56.9	79.1	6.6	NA	NA	10 121	798
JPHC1	41 277	1990-1992	21.1	52.0	49.5	75.8	7.4	21.7	21.3	6872	525
JPHC2	53 645	1992-1995	17.9	52.8	54.0	75.7	7.7	21.6	22.4	11 508	885
3Pref Miyagi	27 131	1984-1984	11.9	55.8	56.5	78.4	11.6	23.6	19.8	4974	250
Miyagi cohort	38 774	1990-1990	16.5	45.2	51.5	81.5	11.1	22.9	21.8	4584	377
Ohsaki	44 704	1994-1994	11.4	52.3	60.0	78.7	8.7	23.3	19.8	7992	542
LSS	45 930	1963-1993	13.9	58.1	60.5	86.2	16.1	NA	18.7	23 832	1099
Takayama study	26 627	1992-1992	14.0	52.3	54.4	83.3	17.4	NA	17.3	4587	264
Korea											
KMCC	11 551	1993-2004	13.6	62.0	56.0	77.7	8.0	23.9	17.4	2274	202
Seoul Male	12 447	1992-1993	15.6	0	49.0	77.7	NA	22.1	18.1	762	82
Singapore											
SCHS	57 714	1993-1999	11.7	56.1	56.1	57.1	8.4	20.3	16.1	8234	874
Taiwan											
CBCSP	20 179	1991-1992	15.2	49.8	49.3	56.2	0.9	NA	NA	2423	206
CVDFACTS	3650	1990-1993	14.8	54.9	53.9	54.2	1.4	25.6	15.5	715	45
India											
Mumbai	143 218	1991-1997	5.3	40.9	50.7	31.3	0.4	22.6	6.8	11 275	68
Total	1 002 258	1963-2006	11.7	51.1	54.6	65.4	7.8	22.8	16.5	144 366	9158

Abbreviations: CBCSP, Community-Based Cancer Screening Project; CHEFS, China National Hypertension Survey Epidemiology Follow-up Study; CVDFACTS, Cardiovascular Disease Risk Factor Two-Township Study; IPHS, Ibaraki Prefectural Health Study; JACC, Japan Collaborative Cohort Study; JPHC1, Japan Public Health Center–Based Prospective Study (initiated in 1990); JPHC2, Japan Public Health Center–Based Prospective Study (initiated in 1992); KMCC, Korean Multi-center Cancer Cohort Study; LSS, Life Span Study; Mumbai, Mumbai Cohort Study; NA, not available; Ohsaki, Ohsaki National Health Insurance Cohort Study; SCHS, Singapore Chinese Health Study; SCS, Shanghai Cohort Study; Seoul Male, Seoul Male Cancer Cohort; SMHS, Shanghai Men’s Health Study; SWHS, Shanghai Women’s Health Study; 3Pref Aichi, Three Prefecture Cohort Study Aichi; 3Pref Miyagi, Three Prefecture Cohort Study Miyagi.

^a^ Including only participants eligible for the current analysis.

^b^ Mean age at enrollment in the baseline (smoking) survey.

^c^ Mean age at starting smoking among ever smokers.

^d^ Mean number of cigarettes smoked per day among current smokers.

**Figure 1.**
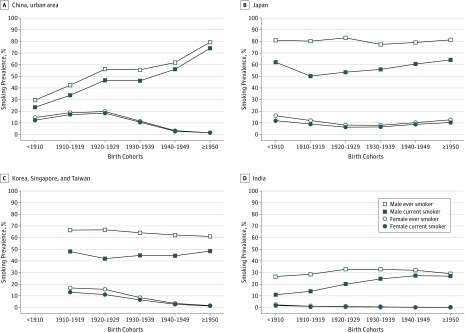
Tobacco Smoking Prevalence by Birth Cohorts and Study Populations

Smoking prevalence varied substantially between men and women and across birth cohorts and countries or regions (Figure 1; eFigure 2 in the Supplement). Men born in the 1920s showed the highest rates of ever smoking in every country except China: 82.9% in Japan; 66.7% in Korea, Singapore, and Taiwan; and 32.8% in India. In China, the rate of ever smoking for men continued to increase in successive birth cohorts: among men born in 1950 or later, 79.4% of those in urban areas and 74.3% of those in rural areas were ever smokers. The difference between current and ever smoking (ie, the rate of cessation) appeared to be much larger for men in Japan and Korea, Singapore, and Taiwan than for those living in China and India. The prevalence of smoking in women remained very low in all Asia Cohort Consortium populations and tended to decline in recent birth cohorts for women living in urban China and Korea, Singapore, and Taiwan although not in rural China, Japan, and India.

The mean age of smoking initiation and mean cigarette consumption per day varied across birth cohorts and countries or regions (eFigure 2, eFigure 3, and eFigure 4 in the Supplement). All populations except women living in urban China and Korea, Singapore, and Taiwan tended to start smoking at a younger age in more recent birth cohorts than in earlier ones. In all Asian populations except urban Chinese women, the mean number of cigarettes smoked per day continued to increase in successive birth cohorts. Japanese men appeared to smoke more cigarettes per day than those living in other Asian countries. When we compared the mean age at quitting smoking stratified by baseline age groups and birth cohorts (eTable 3 in the Supplement), male smokers in recent birth cohorts tended to quit smoking at a younger age irrespective of age groups and countries or regions.

Asian male smokers had a higher risk for all-cause and lung cancer mortality than never smokers, and the risk increased in each successive birth cohort (Table 2). Current smoking was associated with an increasing risk of death, with HRs for all-cause mortality of 1.26 (95% CI, 1.17-1.37) for the pre-1920 birth cohort, 1.47 (95% CI, 1.35-1.61) for the 1920s birth cohort, and 1.70 (95% CI, 1.57-1.84) for the cohort born in 1930 or later. The HRs for lung cancer mortality were 3.38 (95% CI, 2.25-5.07) for the pre-1920 birth cohort, 4.74 (95% CI, 3.56-6.32) for the 1920s birth cohort, and 4.80 (95% CI, 3.71-6.19) for the cohort born in 1930 or later. When stratified by countries or regions, however, China and Korea, Singapore, and Taiwan experienced the highest HRs for lung cancer mortality in the 1920s birth cohort (China: HR, 4.74; 95% CI, 3.71-6.07; and Korea, Singapore, and Taiwan: HR, 4.93; 95% CI, 1.57-15.52). Some participants may not have reached ages at which the adverse effects of smoking are fully realized. Thus, we further evaluated the association stratified by attained age (eTable 4 in the Supplement). When considering participants’ attained age, male smokers who were born in 1930 or later showed increased relative risks for all-cause and lung cancer mortality in all age groups and all countries or regions.

**Table 2.** Risk of Death Associated With Tobacco Smoking by Birth Cohorts in Asian Male Populations

Study Population	Participants, No.	Birth Cohort <1920		Birth Cohort 1920-1929		Birth Cohort ≥1930	
		Deaths, No.	HR (95% CI)^a^	Deaths, No.	HR (95% CI)^a^	Deaths, No.	HR (95% CI)^a^
**Death From All Causes**							
All populations							
Never smoker	169 444	5212	1 [Reference]	6213	1 [Reference]	9646	1 [Reference]
Ever smoker	320 843	15 986	1.22 (1.14-1.30)	19 786	1.37 (1.29-1.46)	28 884	1.59 (1.50-1.68)
Current smoker	249 241	11 587	1.26 (1.17-1.37)	14 299	1.47 (1.35-1.61)	23 468	1.70 (1.57-1.84)
By study populations: current smoker vs never smoker							
China–urban area	107 265	1223	1.09 (0.97-1.23)	4168	1.48 (1.39-1.58)	6195	1.52 (1.44-1.61)
Japan	162 259	13 207	1.38 (1.32-1.45)	10 907	1.63 (1.47-1.80)	16 243	1.89 (1.81-1.97)
Korea, Singapore, and Taiwan	43 981	210	1.14 (0.83-1.58)	2139	1.31 (1.01-1.69)	4863	1.74 (1.57-1.94)
India	79 705	1059	1.09 (0.92-1.28)	1981	1.18 (1.06-1.30)	4242	1.45 (1.36-1.54)
**Death From Lung Cancer**^b^							
All populations							
Never smoker	169 444	93	1 [Reference]	197	1 [Reference]	346	1 [Reference]
Ever smoker	320 843	962	3.00 (2.24-4.02)	2043	3.77 (2.94-4.84)	3133	4.09 (3.26-5.15)
Current smoker	249 241	765	3.38 (2.25-5.07)	1705	4.74 (3.56-6.32)	2797	4.80 (3.71-6.19)
By study populations: current smoker vs never smoker							
China–urban area	107 265	48	2.69 (1.46-4.96)	485	4.74 (3.71-6.07)	913	4.32 (2.62-7.13)
Japan	162 259	780	4.26 (3.25-5.58)	1074	5.22 (4.01-6.79)	1499	6.08 (4.40-8.41)
Korea, Singapore, and Taiwan	43 981	12	NA^c^	259	4.93 (1.57-15.52)	601	4.42 (2.27-8.61)

Abbreviations: HR, hazard ratio; NA, not available.

^a^ Adjusted for age, educational level, marital status, rural or urban residence, and body mass index and stratified by 5-year groups of birth year and enrollment year.

^b^ The number of deaths from lung cancer was less than 20 for any of the birth cohorts included in the analysis for Indians; thus, no HR was estimated.

^c^ Not estimated because of small sample size (<20 events).

**Figure 2.**
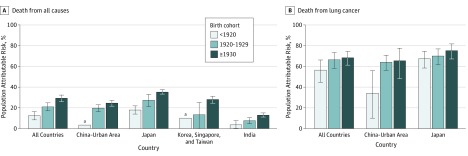
Population-Attributable Risk of Tobacco Smoking in Asian Male Populations Error bars indicate 95% CIs for the population-attributable risk estimates. Owing to small sample sizes and unstable estimates by birth cohorts, the population-specific, population-attributable risks for all-cause mortality and lung cancer mortality are not reported for India and Korea, Singapore, and Taiwan. ^a^95% CI of population attributable risk includes zero owing to small sample sizes and unstable estimates of the hazard ratio.

Smoking at an earlier age and smoking more cigarettes per day were each associated, in a dose-response manner, with increased relative risks for all-cause and lung cancer mortality among men (eTable 5 in the Supplement). These associations were seen in all birth cohorts and were stronger in the most recent birth cohort. On the other hand, men who quit smoking before age 40 years showed no elevated risk of all-cause mortality compared with never smokers (eTable 6 in the Supplement). In the cohort born in 1930 or later, however, those who quit smoking before age 40 years had a 1.48-fold increased risk of lung cancer mortality. Tobacco smoking accounted for a substantial portion of the deaths of Asian men (Figure 2). Overall, smoking-associated PARs for all-cause mortality increased from 12.5% (95% CI, 8.4%-16.3%) in the pre-1920 birth cohort to 21.1% (95% CI, 17.3%-24.9%) in the 1920s birth cohort to 29.3% (95% CI, 26.0%-32.3%) in the cohort born in 1930 or later. This pattern was consistently observed in all countries and regions. Among Asian men, the smoking-associated PARs for lung cancer mortality were 56.6% (95% CI, 44.7%-66.3%) in the pre-1920 cohort, 66.6% (95% CI, 58.3%-73.5%) in the 1920s cohort, and 68.4% (95% CI, 61.3%-74.4%) in the cohort born in 1930 or later.

Compared with their male counterparts, few Asian women smoked cigarettes regularly. Nevertheless, we observed similar patterns of the association described for men—more recent birth cohorts showed stronger associations of smoking with all-cause and lung cancer mortality. Current smoking was associated with an increasing risk of death, with HRs for all-cause mortality of 1.29 (95% CI, 1.22-1.35) for the pre-1920 birth cohort, 1.52 (95% CI, 1.44-1.62) for the 1920s birth cohort, and 1.71 (95% CI, 1.57-1.86) for the cohort born in 1930 or later. The HRs for lung cancer mortality were 2.94 (95% CI, 2.33-3.71) for the pre-1920 birth cohort, 4.17 (95% CI, 3.25-5.36) for the 1920s birth cohort, and 3.53 (95% CI, 2.99-4.16) for the cohort born in 1930 or later (Table 3). However, we found different country-specific or region-specific risk patterns for lung cancer mortality among women. In urban China and Korea, Singapore, and Taiwan, the highest relative risk for lung cancer mortality was found in the 1920s birth cohort, even in the stratified analyses by attained age; however, this risk pattern was not seen in Japan. A dose-response association of initiation age and daily cigarette consumption with mortality was consistently found for women (eTable 7 in the Supplement). Smoking-associated PARs for all-cause and lung cancer mortality were considerably lower than those observed among men (eFigure 5 in the Supplement). Sensitivity analyses using different adjustment variables (excluding body mass index or additionally adjusting for physical activity) or a different imputation method (applying multiple imputation if the proportion of missing covariates was >3%) yielded similar results (eTables 8-10 in the Supplement).

**Table 3.** Risk of Death Associated With Tobacco Smoking by Birth Cohorts in Asian Female Populations

Study Population	Participants, No.	Birth Cohort <1920		Birth Cohort 1920-1929		Birth Cohort ≥1930	
		Deaths, No.	HR (95% CI)^a^	Deaths, No.	HR (95% CI)^a^	Deaths, No.	HR (95% CI)^a^
**Death From All Causes**							
All populations							
Never smoker	472 081	17 151	1 [Reference]	14 532	1 [Reference]	18 946	1 [Reference]
Ever smoker	39 890	3158	1.26 (1.22-1.31)	2345	1.49 (1.42-1.56)	2507	1.66 (1.55-1.79)
Current smoker	33 719	2472	1.29 (1.22-1.35)	1956	1.52 (1.44-1.62)	2173	1.71 (1.57-1.86)
By study populations: current smoker vs never smoker							
China–urban area	106 450	1285	1.23 (1.06-1.41)	1723	1.41 (1.25-1.59)	5820	1.58 (1.43-1.74)
Japan	259 947	16 324	1.31 (1.25-1.38)	10 750	1.57 (1.47-1.69)	9706	1.80 (1.64-1.99)
Korea, Singapore, and Taiwan	50 775	261	1.23 (0.67-2.26)	2147	1.62 (1.44-1.82)	3059	1.90 (1.68-2.15)
India	58 474	553	0.93 (0.44-1.99)	925	1.28 (0.68-2.38)	1539	1.30 (0.72-2.36)
**Death From Lung Cancer**^b^							
All populations							
Never smoker	472 081	289	1 [Reference]	437	1 [Reference]	1059	1 [Reference]
Ever smoker	39 890	129	2.87 (2.22-3.71)	217	3.85 (3.04-4.87)	253	3.21 (2.77-3.72)
Current smoker	33 719	104	2.94 (2.33-3.71)	186	4.17 (3.25-5.36)	233	3.53 (2.99-4.16)
By study populations: current smoker vs never smoker							
China–urban area	106 450	41	1.96 (0.99-3.88)	103	5.02 (3.25-7.74)	558	3.00 (2.30-3.91)
Japan	259 947	321	3.12 (2.42-4.03)	350	3.72 (2.85-4.86)	441	3.78 (2.92-4.88)
Korea, Singapore, and Taiwan	50 775	12	NA^c^	134	6.92 (4.72-10.1)	242	4.26 (3.03-6.00)

Abbreviations: HR, hazard ratio; NA, not available.

^a^ Adjusted for age, educational level, marital status, rural or urban residence, and body mass index and stratified by 5-year groups of birth year and enrollment year.

^b^ The number of deaths from lung cancer was less than 20 for any of the birth cohorts included in the analysis for Indians; thus, no HR was estimated.

^c^ Not estimated because of small sample size (<20 events).

## Discussion

A previous analysis of data from the Asia Cohort Consortium estimated that tobacco smoking might have been responsible for the deaths of approximately 2 million Asian individuals in 2004.^4^ In the present study, we found that Asian men in recent birth cohorts tended to start smoking at a younger age and smoked more cigarettes per day than those born in an earlier era. Consequently, smokers born in 1930 or later were at a higher relative risk of all-cause and lung cancer mortality than were smokers born before 1920. For Asian men born in 1930 or later, tobacco smoking was associated with 29.3% of total deaths and 68.4% of lung cancer deaths, rates that were much higher than the proportions for those born before 1920 (all-cause mortality, 12.5%, and lung cancer mortality, 56.6%). These estimates suggest that most Asian countries are still in the early stages of the tobacco smoking epidemic and that tobacco smoking will remain a major public health threat in the coming decades.

With the tobacco smoking epidemic worsening in the past few decades, we found that the relative risk of death attributable to tobacco smoking continued to rise in successive birth cohorts in Asia. Similar results were also reported from previous studies conducted in Asia^11,12,13^ and Western countries.^19,20^ Changes in patterns of tobacco use may be involved in the risk increments; however, it is also possible that other factors may contribute, in part, to the increased relative risks in successive birth cohorts.

Although the HRs for all-cause and lung cancer deaths associated with tobacco smoking continued to rise in most Asian populations, these risk estimates remained lower than those reported in Western countries. Studies in Western countries reported an almost 3-fold elevated risk for all-cause mortality and an approximately 20-fold elevated risk for lung cancer mortality among current smokers compared with never smokers.^19,21,22,23,24,25,26,27^ However, we report HRs for all-cause mortality of 1.70 and lung cancer mortality of 4.80 in the cohort of men born in 1930 or later. These HRs are comparable with previous reports from Asia.^11,12^ Our risk estimates are also similar to those observed for low-intensity smokers among the US population^28^ and in the early follow-up years of the British Doctors Study.^24^

Asian men showed a late initiation and lower smoking intensity than their Western counterparts. In the United States, men born in the 1920s, on average, began smoking before age 18 years.^28^ Similarly, German men born between 1926 and 1930 started smoking at age 18 years.^29^ Despite the trend of earlier initiation, we found that the mean age at smoking initiation was still later than age 20 years in Asian men, even in the most recent birth cohort (≥1950). Furthermore, mean daily cigarette consumption remained under 20 in most Asian male smokers (7 cigarettes in India and approximately 17 cigarettes in China and Korea, Singapore, and Taiwan) except in Japan. This number is substantially lower than those reported in US male smokers born in the 1930s, which peaked at approximately 25 cigarettes per day during their midlife.^30^ Our findings indicate that many Asian countries may still be in the early stages of the tobacco epidemic or show a relatively lower degree of smoking than Western countries. Antismoking campaigns are imperative to stop the upward trend in the tobacco smoking epidemic and reduce the burden of tobacco smoking in Asia.

On the other hand, our modest risk estimates for lung cancer mortality may reflect the possibility that, in Asia, other background risk factors (ie, outdoor and household air pollution and secondhand smoke) are intimately involved in lung cancer mortality.^31,32,33^ These background risk factors may increase the risk of lung cancer among never smokers. A previous study has reported that age-standardized lung cancer mortality was much higher for lifelong never smokers living in Asia than in other racial/ethnic groups.^31^ Despite a notably low prevalence of smoking, Asian women (especially Chinese women) showed a higher rate of lung cancer than Western women,^31^ suggesting a high likelihood of death from lung cancer among never smokers. If the tobacco epidemic across Asian populations persists or grows steadily, most Asian countries will face the double burden of lung cancer attributable to both tobacco smoking and other background risk factors. It is also possible that the full effect of tobacco smoking may increase more dramatically than we have projected because of potential interaction between tobacco smoking and other lung cancer risk factors that are prevalent in Asia.

Our study suggests that tobacco control interventions may have started to affect the tobacco epidemic in some Asian populations: male smokers in the most recent birth cohort tended to quit smoking at younger ages. However, the overall cessation rates vary by countries or regions. Cessation rates remain relatively low in China and India (<10%) but are more than 20% in Japan and Korea, Singapore, and Taiwan, suggesting differences in the effectiveness of antismoking campaigns or the availability of smoking cessation services across Asia. In this study, earlier cessation is associated with a reduced risk of all-cause mortality. No appreciable excess risk of all-cause mortality was found among smokers who stopped smoking before age 40 years, which is in line with previous reports.^21,23^ Cessation is the most effective means to reduce smoking-induced health burdens. Population-specific intervention strategies need to be developed and more fully implemented in all Asian countries, especially low- and middle-income countries and regions.

For Asian women, the prevalence of smoking remains very low, but smoking behaviors vary by countries and regions, resulting in different patterns of lung cancer mortality. In urban China and Korea, Singapore, and Taiwan, women in recent birth cohorts showed a declining trend in smoking prevalence, a delayed initiation of regular smoking, and a decline or plateau in the mean number of cigarettes smoked per day. These differences may explain the reduced relative risk of lung cancer mortality in the recent birth cohorts of these 2 populations. Similarly, a previous study reported a decrease in smoking prevalence among Chinese women in recent birth cohorts.^11^ However, we found that, despite the favorable changes in smoking behaviors, the relative risk of overall mortality continued to rise in successive birth cohorts. Although this study includes more than a half-million Asian women, the number of female smokers is still too limited to reliably quantify the associations between smoking and mortality. Continued follow-up of the study participants should provide additional data for future analyses of these associations.

### Strengths and Limitations

To our knowledge, this study is the largest prospective investigation of birth cohort–specific and country-specific or region-specific smoking patterns and their association with mortality in Asian populations. By using long-term follow-up data from 20 cohorts conducted in different periods and multiple countries and regions at different stages of economic development, this study provides a more detailed picture on the pattern of the tobacco epidemic and consequent mortality in Asia than that provided by any single cohort study conducted in a single country. However, there are a few limitations of the study. First, our participants may not be a representative sample for each of the 7 countries or regions; thus, the smoking patterns we described may not entirely represent the patterns in those countries or regions. However, our results present similar patterns of the association and comparable risk estimates with those reported in recent nationwide surveys.^11,13,34^ Second, we had no data on secondhand smoke and use of smokeless tobacco. The lack of information on these tobacco exposures might affect the association between smoking and mortality. In addition, some other confounding variables were not adjusted in this study; thus, potential residual confounding could affect our risk estimates. Third, because smoking data were collected only at baseline, we could not consider possible changes in smoking behaviors over time. Fourth, it is possible that the adverse effects of smoking have not been fully potentiated in more recent birth cohorts. Fifth, we could not quantify the association of smoking and mortality in contemporary birth cohorts. Based on the smoking characteristics presented in this study, however, we project that the magnitude of the association of smoking and mortality in contemporary birth cohorts is likely to be greater than what we presented for the cohort of those born in 1930 or later, particularly given the limited antismoking campaigns in many low- and middle-income Asian countries and regions.

## Conclusions

Asia has now become the center of the global tobacco epidemic and inevitably will face a growing burden of tobacco-related health problems. An increasing proportion of men are smoking, starting at a younger age, and smoking more heavily. This finding suggests a continuing increase in the risk of death due to tobacco smoking. It is possible that we will also see further increases in smoking among women in certain Asian countries. As recommended by the World Health Organization Framework Convention on Tobacco Control,^5^ all Asian countries should implement comprehensive tobacco control policies, such as raising tobacco taxes and prices, implementing smoke-free laws and bans on advertising and promotion, providing cessation assistance, and using warning labels on tobacco packages, to end the tobacco epidemic in Asia. For current smokers, quitting as soon as possible is the best strategy to reduce the risk associated with smoking.

## Notes

Supplement. **eAppendix.** Supplemental Methods **eReferences** **eFigure 1.** 20 Participating Cohorts for the Current Pooled Analysis **eFigure 2.** Tobacco Smoking Patterns in Rural China Populations **eFigure 3.** Mean Age at Starting Tobacco Smoking by Birth Cohorts and Study Populations **eFigure 4.** Mean Number of Cigarettes Smoked per Day by Birth Cohorts and Study Populations **eFigure 5.** Population Attributable Risk of Tobacco Smoking in Asian Female Populations **eTable 1.** Descriptions of Participating Cohorts **eTable 2.** Baseline Characteristics Across Birth Cohorts **eTable 3.** Mean Age at Quitting Tobacco Smoking Among Former Smokers by Birth Cohorts and Study Populations: Asian Male Populations **eTable 4.** Risk of Death Associated With Tobacco Smoking by Birth Cohorts and Attained Age in Asian Male Populations: Current vs Never Smokers **eTable 5.** Risk of Death Associated With Tobacco Smoking History by Birth Cohorts in Asian Male Populations **eTable 6.** Risk of Death Associated With Age at Quitting Smoking by Birth Cohorts in Asian Male Populations **eTable 7.** Risk of Death Associated With Tobacco Smoking History by Birth Cohorts in Asian Female Populations **eTable 8.** Risk of Death Associated With Tobacco Smoking by Birth Cohorts in Asian Populations: Sensitivity Analysis Not Adjusted for Body Mass Index **eTable 9.** Risk of Death Associated With Tobacco Smoking by Birth Cohorts in Asian Populations: Sensitivity Analysis Further Adjusted for Physical Activity Status **eTable 10.** Risk of Death Associated With Tobacco Smoking by Birth Cohorts in Asian Populations: Sensitivity Analysis Using the Multiple Imputation Method
