# Supplementary material for: Tobacco Smoking and Mortality in Asia: A Pooled Meta-analysis
Source: JAMA Netw Open. 2019 Mar 29;2(3):e191474. doi: 10.1001/jamanetworkopen.2019.1474 (PMC6450311; doi:10.1001/jamanetworkopen.2019.1474)
Supplement: Supplement. — eAppendix. Supplemental Methods eReferences eFigure 1. 20 Participating Cohorts for the Current Pooled Analysis eFigure 2. Tobacco Smoking Patterns in Rural China Populations eFigure 3. Mean Age at Starting Tobacco Smoking by Birth Cohorts and Study Populations eFigure 4. Mean Number of Cigarettes Smoked per Day by Birth Cohorts and Study Populations eFigure 5. Population Attributable Risk of Tobacco Smoking in Asian Female Populations eTable 1. Descriptions of Participating Cohorts eTable 2. Baseline Characteristics Across Birth Cohorts eTable 3. Mean Age at Quitting Tobacco Smoking Among Former Smokers by Birth Cohorts and Study Populations: Asian Male Populations eTable 4. Risk of Death Associated With Tobacco Smoking by Birth Cohorts and Attained Age in Asian Male Populations: Current vs Never Smokers eTable 5. Risk of Death Associated With Tobacco Smoking History by Birth Cohorts in Asian Male Populations eTable 6. Risk of Death Associated With Age at Quitting Smoking by Birth Cohorts in Asian Male Populations eTable 7. Risk of Death Associated With Tobacco Smoking History by Birth Cohorts in Asian Female Populations eTable 8. Risk of Death Associated With Tobacco Smoking by Birth Cohorts in Asian Populations: Sensitivity Analysis Not Adjusted for Body Mass Index eTable 9. Risk of Death Associated With Tobacco Smoking by Birth Cohorts in Asian Populations: Sensitivity Analysis Further Adjusted for Physical Activity Status eTable 10. Risk of Death Associated With Tobacco Smoking by Birth Cohorts in Asian Populations: Sensitivity Analysis Using the Multiple Imputation Method [file jamanetwopen-2-e191474-s001.pdf]

Supplementary Online Content

Yang JJ, Yu D, Wen W, et al. Tobacco smoking and mortality in Asia: a pooled meta-analysis. *JAMA Netw Open.* 2019;2(3):e191474. doi:10.1001/jamanetworkopen.2019.1474

eAppendix. Supplemental Methods
eReferences
eFigure 1. 20 Participating Cohorts for the Current Pooled Analysis
eFigure 2. Tobacco Smoking Patterns in Rural China Populations
eFigure 3. Mean Age at Starting Tobacco Smoking by Birth Cohorts and Study Populations
eFigure 4. Mean Number of Cigarettes Smoked per Day by Birth Cohorts and Study Populations
eFigure 5. Population Attributable Risk of Tobacco Smoking in Asian Female Populations
eTable 1. Descriptions of Participating Cohorts
eTable 2. Baseline Characteristics Across Birth Cohorts
eTable 3. Mean Age at Quitting Tobacco Smoking Among Former Smokers by Birth Cohorts and Study Populations: Asian Male Populations
eTable 4. Risk of Death Associated With Tobacco Smoking by Birth Cohorts and Attained Age in Asian Male Populations: Current vs Never Smokers
eTable 5. Risk of Death Associated With Tobacco Smoking History by Birth Cohorts in Asian Male Populations
eTable 6. Risk of Death Associated With Age at Quitting Smoking by Birth Cohorts in Asian Male Populations
eTable 7. Risk of Death Associated With Tobacco Smoking History by Birth Cohorts in Asian Female Populations
eTable 8. Risk of Death Associated With Tobacco Smoking by Birth Cohorts in Asian Populations: Sensitivity Analysis Not Adjusted for Body Mass Index
eTable 9. Risk of Death Associated With Tobacco Smoking by Birth Cohorts in Asian Populations: Sensitivity Analysis Further Adjusted for Physical Activity Status
eTable 10. Risk of Death Associated With Tobacco Smoking by Birth Cohorts in Asian Populations: Sensitivity Analysis Using the Multiple Imputation Method

This supplementary material has been provided by the authors to give readers additional information about their work.

© 2019 Yang JJ et al. *JAMA Network Open.*

eAppendix. Supplemental Methods

Data Harmonization

For use of the ACC collaborative project data, each principal investigator should submit his/her study proposal to the ACC Coordinating Center. Study proposals are reviewed by the ACC executive committee. Along with an executive committee's approval, the ACC Coordinating Center starts to consolidate the data for the newly initiated project. First, the ACC Coordinating Center invites cohort studies to participate in the project; then, participating cohorts submit all relevant data, including the most recent follow-up information, if available. After data collection from all participating cohorts, the ACC Coordinating Center conducts data updating, cleaning, and pooling—the final dataset is tailored for each specific project only. Once the dataset is ready, each principal investigator submits the Data Use Agreement Form and the Remote Access Application Form to the ACC Coordinating Center. Based on information in the Remote Access Application Form (i.e., IP address and a proxy server), the ACC Coordinating Center opens a remote access account for each principal investigator—the investigator can access the ACC Data Center via Virtual Private Network and conduct data analysis using datasets and the data dictionary provided by the ACC Coordinating Center.

Random-effects Meta-analysis

For the random-effects meta-analysis, we assumed that the effect of tobacco smoking on mortality was cohort-specific: under this assumption, the log-HR for tobacco smoking has a fixed-effect component which is common to all cohorts within each country and a cohort-specific random-effect. Random-effects for the log-HRs were assumed to be normally distributed, with a mean value of zero. Specifically, $\hat{\beta}_{ij}$, the estimated log-HR for the *j*-th

© 2019 Yang JJ et al. *JAMA Network Open.*

smoking exposure level in the *i*-th cohort, follows the distribution $\hat{\beta}_{ij} \sim N(\beta_j, \hat{\sigma}_{ij}^2 + \hat{\tau}_j^2)$, where $\hat{\sigma}_{ij}^2$ is the within-study variance of $\hat{\beta}_{ij}$ and $\hat{\tau}_j^2$ is the between-cohort variance of $\hat{\beta}_{ij}$, as estimated by the Cox regression model.[1,2] We estimated the β_j parameters and their 95% CIs in the meta-analysis using SAS version 9.3 (SAS Institute Inc., Cary, North Carolina, USA).

eReferences.

1. DerSimonian R, Laird N. Meta-analysis in clinical trials. Control Clin Trials. 1986 Sep;7(3):177–88.

2. Brockwell SE, Gordon IR. A comparison of statistical methods for meta-analysis. Stat Med. 2001 Mar 30;20(6):825–40.

© 2019 Yang JJ et al. *JAMA Network Open.*

eFigure 1. 20 Participating Cohorts for the Current Pooled Analysis

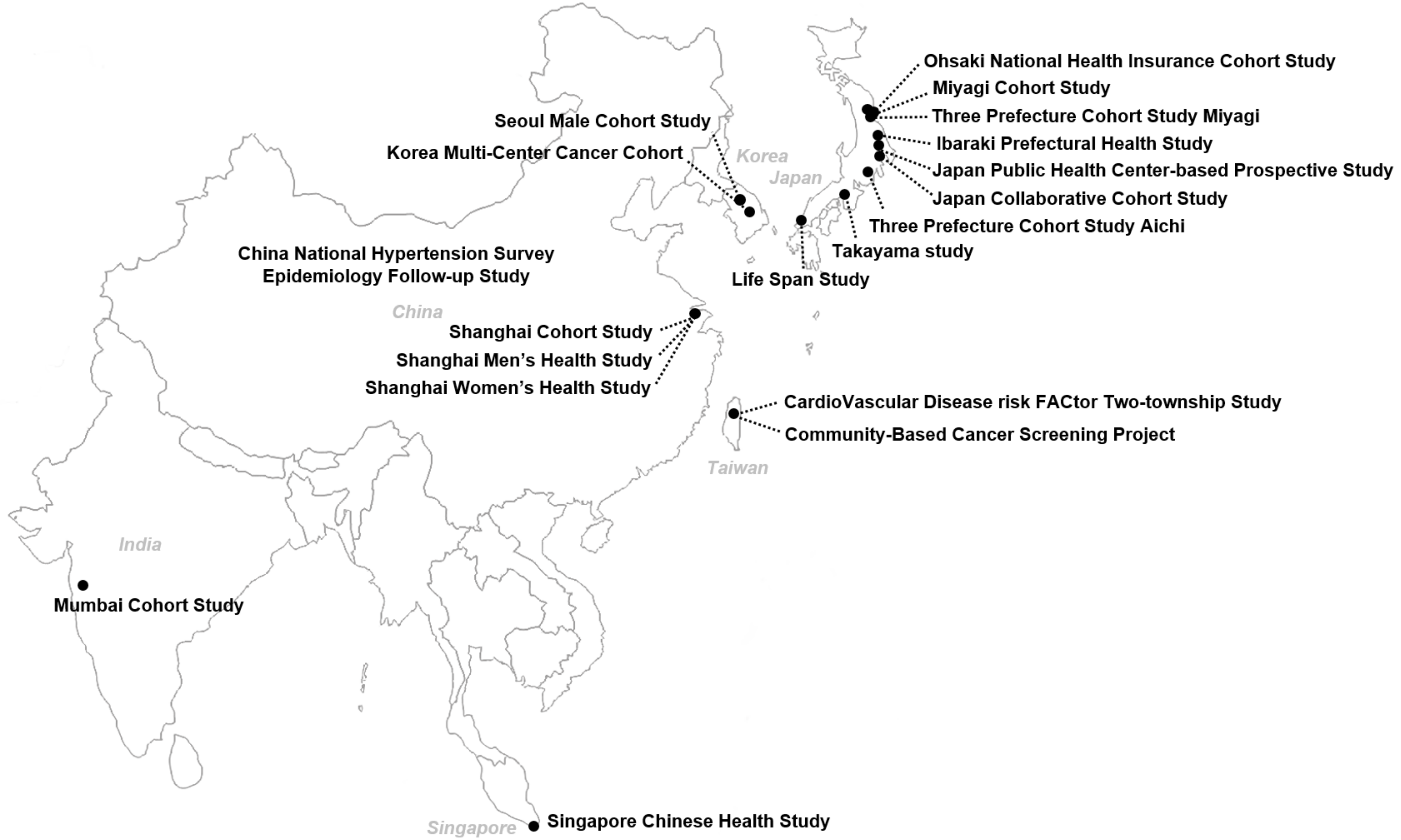

© 2019 Yang JJ et al. *JAMA Network Open.*

eFigure 2. Tobacco Smoking Patterns in Rural China Populations

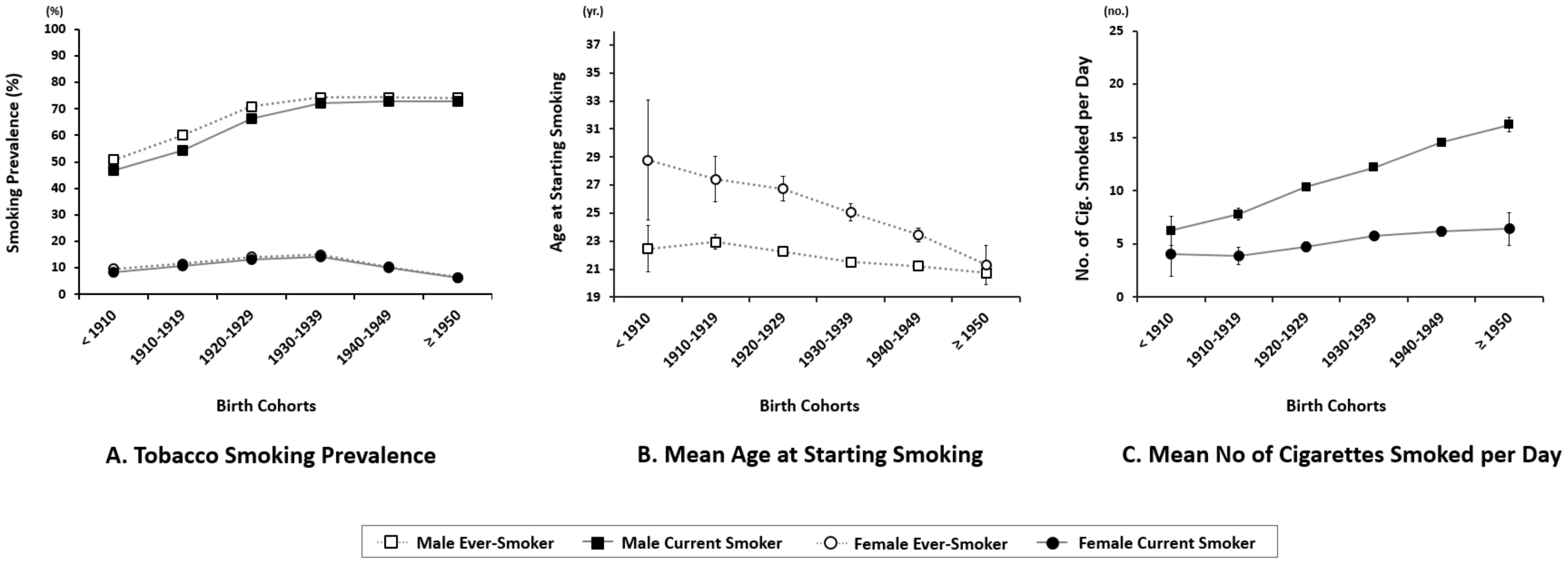

© 2019 Yang JJ et al. *JAMA Network Open.*

eFigure 3. Mean Age at Starting Tobacco Smoking by Birth Cohorts and Study Populations

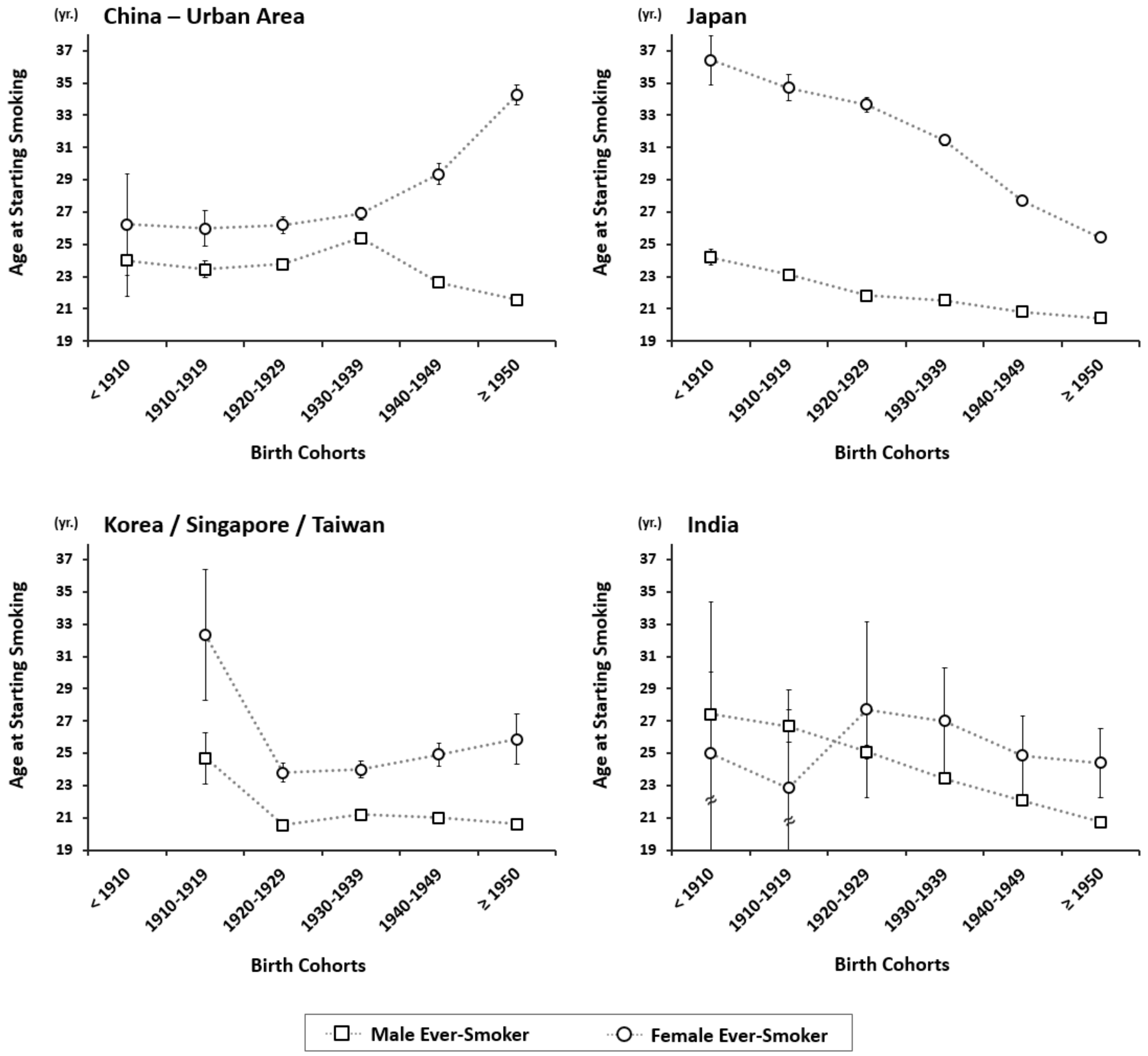

© 2019 Yang JJ et al. *JAMA Network Open.*

eFigure 4. Mean Number of Cigarettes Smoked per Day by Birth Cohorts and Study Populations

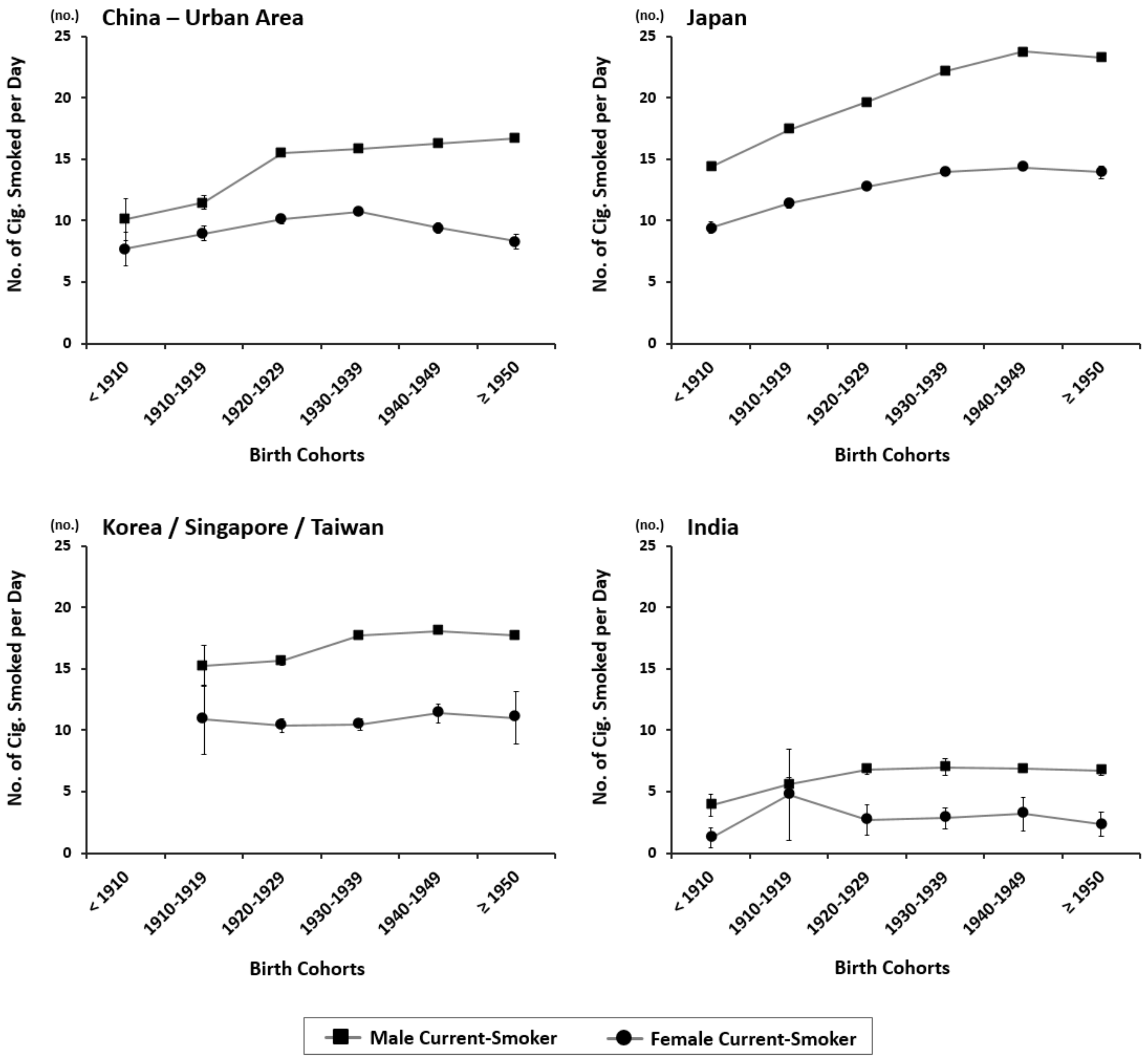

© 2019 Yang JJ et al. *JAMA Network Open.*

eFigure 5. Population Attributable Risk of Tobacco Smoking in Asian Female Populations

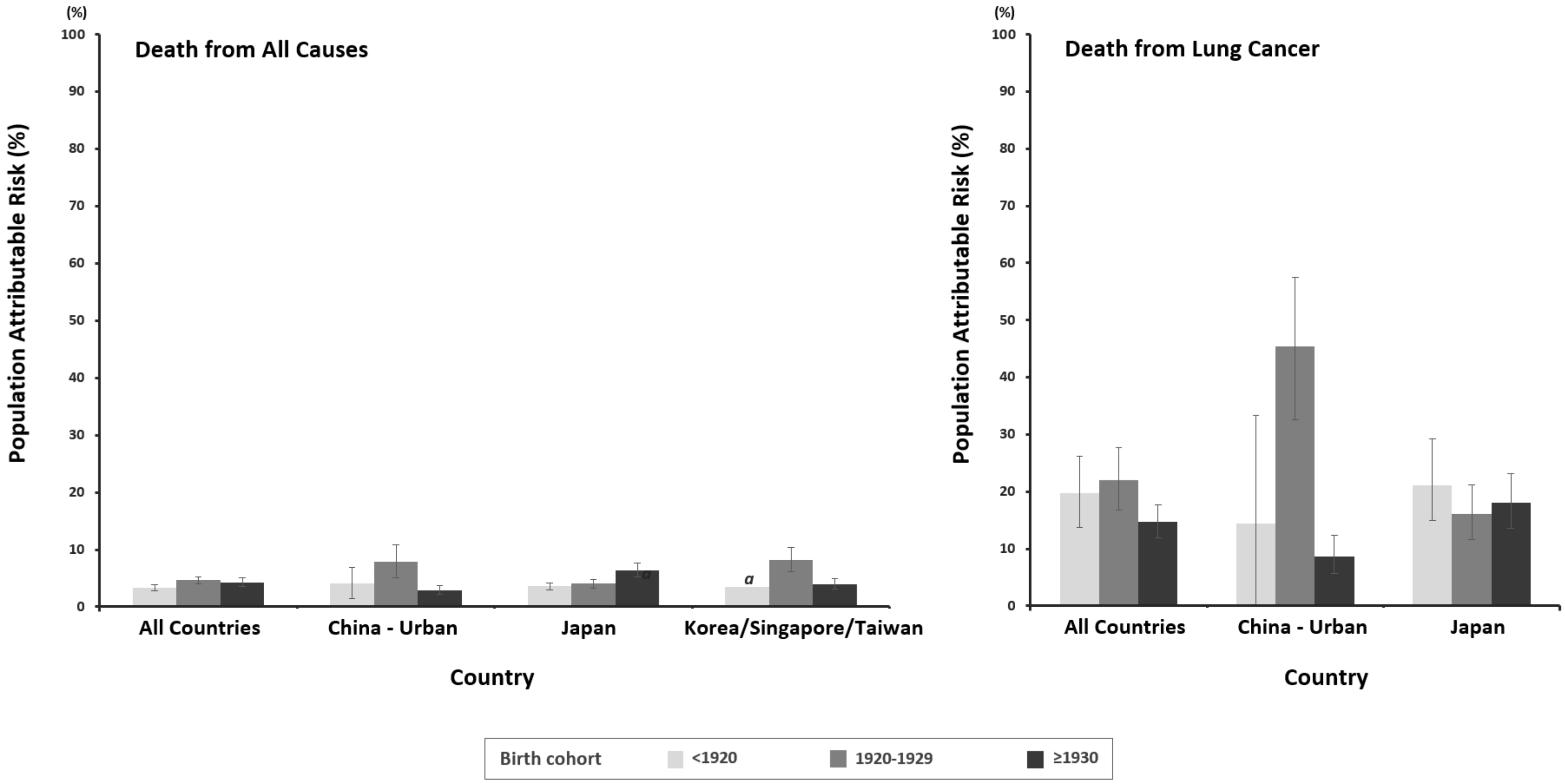

[a] 95% CI of population attributable risk includes zero due to small sample sizes and unstable estimates of the hazard ratio.
Error bars indicate 95% CIs for the population attributable risk estimates. Due to small sample sizes and unstable estimates by birth cohorts, the population-specific population attributable risks for all-cause mortality and lung-cancer mortality are not reported for India and Korea/Singapore/Taiwan and India.

© 2019 Yang JJ et al. *JAMA Network Open.*

eTable 1. Descriptions of Participating Cohorts

Cohort Name (Abbreviation)	Descriptions	Reference
China National Hypertension Survey Epidemiology Follow-up Study (CHEFS)	• This study is based on the China National Hypertension Survey in 1991, which included a nationally representative sample of the general population from all 30 provinces, autonomous regions, and municipalities of mainland China. Between 1999-2000, the China National Hypertension Survey Epidemiology Follow-up Study was conducted in 17 provinces. Using a standard questionnaire, baseline information was collected at one clinic visit by trained physicians and nurses. In-person follow-up interviews for all study participants were conducted to ascertain vital status and disease incidence. To identify cases for cardiovascular diseases and renal disorders, medical records and death certificates were reviewed. The follow-up rate was 95% over the first eight years—follow-up ended in 2000.	Lancet.2009;374(9703):1765-72. doi: 10.1016/S0140-6736(09)61199-5.
Shanghai Cohort Study (SCS)	• Between January 1, 1986 and September 30, 1989, a total of 18,244 men who were aged 45-64 and had no history of cancer were recruited in Shanghai areas. During in-person interviews using a structured questionnaire, baseline information and biological samples were collected. Via data linkages to the Shanghai Cancer Registry and Shanghai Municipal Vital Statistics, as well as in-person visits, disease incidence (i.e., cancer, cardiovascular diseases, etc.) and death were ascertained among study populations.	Int J Cancer. 2009;125(11):2652-9. doi: 10.1002/ijc.24583.
Shanghai Men's Health Study (SMHS)	• Between April 2002 and June 2006, a total of 61,504 cancer-free Chinese men aged 40-74 were enrolled in seven Shanghai communities. Using a validated questionnaire asking about lifestyle and dietary factors, baseline and follow-up surveys were conducted. At baseline, blood and urine samples were obtained from volunteers. Vital status and disease outcomes were regularly updated by data linkages and repeated follow-up surveys. This study is still ongoing.	Int J Epidemiol. 2015;44(3):810-8. doi: 10.1093/ije/dyv013.
Shanghai Women's Health Study (SWHS)	• Between 1997 and 2000, a total of 74,942 women aged 40-70 were enrolled in Shanghai communities, with a 92% response rate. At the baseline survey, all participants completed detailed baseline questionnaires asking dietary and lifestyle information and anthropometrics. About 88% of cohort members donated urine and blood samples. Study populations were followed via biennial in-person recontact and periodic linkage to cancer and vital statistic registries. This study is still ongoing.	Am J Epidemiol. 2005;162(11):1123-31. doi: 10.1093/aje/kwi322
Three Prefecture Cohort Study Aichi (3Pref Aichi)	• In 1985, residents of the Aichi Prefecture (Nagoya City and Inuyama City), aged ≥40 years, were invited to participate in the study. Self-administered questionnaires in sealed envelopes were distributed to all residents aged ≥40, and a total of 33,538 responded to questionnaires (response rate: ~93%). In cases of duplication or not providing basic information, some subjects were excluded from the cohort. Study participants were followed-up for death and cancer incidence through record linkage to residence certificates, death certificates, and local cancer registry data. This study was terminated in 2000.	J Epidemiol. 2017;27(4):193-199. doi: 10.1016/j.je.2016.05.003.
Ibaraki Prefectural Health Study (IPHS)	• In 1993, the Ibaraki prefectural government initiated a community-based cohort study including 98,196 individuals (33,414 men and 64,782 women) aged 40-79. All study populations underwent an annual health checkup in 1993 and followed-up to ascertain deaths. If participants moved out from the Ibaraki prefectural, they were treated as censored. A	Circulation. 2009;119(16):2136-45. doi: 10.1161/CIRCULA

© 2019 Yang JJ et al. *JAMA Network Open.*

		systematic review of death certificates and resident registrations were conducted; and the validation study showed high sensitivity and specificity of this method.	TIONAHA.108.795666.
Japan Collaborative Cohort Study (JACC)	•	A total of 110,585 participants (46,395 men and 64,190 women) from the 45 areas of Japan were included in this study. Baseline information was collected from 1988 through 1990, using a self-administered questionnaire. Investigators reviewed the population registry information of survivors. This study was terminated at the end of 2009: a total of 27,410 deaths were identified during the median follow-up of 18 years. The main cause of death was cancer and circulatory diseases.	J Epidemiol. 2013;23(3):227-32.
The Japan Public Health Center-based Prospective Study (JPHC)	•	Based on 11 public health center areas in Japan, this study was launched in 1990 (JPHC I) and 1993 (JPHC II). A total of 140,420 subjects aged 40-59 were enrolled with a self-administered questionnaire survey. Repeated follow-up surveys were also conducted after 5 and 10 years. Study participants were followed-up for incidence of cancer and cardiovascular diseases and death by data linkage to cancer and residential registries. This study is still ongoing.	J Epidemiol. 2001;11(6 Suppl):S3-7.
Three Prefecture Cohort Study Miyagi (3Pref Miyagi)	•	In 1984, residents of the Miyagi Prefecture (Sendai City and Wakuya/Tajiri Town), aged ≥40 years, were invited to participate in the study. Self-administered questionnaires in sealed envelopes were distributed to all residents aged ≥40, and a total of 31,769 responded to the questionnaires (response rate: ~97%). In cases of duplication or not providing basic information, some subjects were excluded from the cohort. Study participants were followed for death and cancer incidence through record linkage to residence certificates, death certificates, and local cancer registry data. This study was terminated in 1999.	J Epidemiol. 2017;27(4):193-199. doi: 10.1016/j.je.2016.05.003.
Miyagi Cohort Study (Miyagi)	•	In 1990, this study was initiated in 14 municipalities of Miyagi Prefecture, Japan. A total of 47,605 residents aged 40-64 (coverage rate 91.7%: 13,992 men and 17,353 women) participated in this study. Two self-administered questionnaires regarding lifestyle and personality were used to collect baseline information. Through data linkage to cancer and death registries, study participants were followed-up for cancer and death. This study is still ongoing.	J Epidemiol. 2004;14 Suppl 1:S2-6.
Ohsaki National Health Insurance Cohort Study (Ohsaki)	•	In 1995, subjects who were National Health Insurance beneficiaries and received care at the Ohsaki Public Health Center were enrolled in this study. A total of 51,253 men and women aged 40-79 were recruited. Participants were regularly followed-up for death and other diseases via reviews of death certificates and National Health Insurance files. For cancer incidence, data linkage to the local cancer registry was conducted. This study is still ongoing.	J Epidemiol. 1998;8(5):258-63.
Life Span Study (LSS)	•	This study consists of 120,000 persons including atomic bomb survivors, 94,000 of whom were in the city at the time of the bombing. Another 26,000 were age- and sex-matched residents who were not in Hiroshima or Nagasaki at the time of the bombing. This cohort was established based on the 1950 Japanese national census. Participants were followed-up for mortality and cancer incidence. Lifestyle information was collected via a clinical sub-study and mailed questionnaires. For this unique cohort, participants were restricted to those who were exposed to less than 0.1 gray of bomb radiation. This study is still ongoing.	Radiat Res. 2012;177(3):229-43.
Takayama study (Takayama)	•	This study was initiated on September 1, 1992. At the baseline survey, a total of 31,552 Takayama residents, aged ≥35 years (14,427 men and 17,125 women, representing 85.3% of the total population), completed a self-administered questionnaire. Study participants were followed-up for cancer diagnosis, death, or emigration. The most recent follow-up was completed in 2008.	Nutr Cancer. 2018 Nov 20:1-7. doi: 10.1080/01635581.2018.1512638.

© 2019 Yang JJ et al. *JAMA Network Open.*

Korea Multi-Center Cancer Cohort<br>(KMCC)	• Between 1993 and 2004, 19,688 men and women over 18 years of age were recruited from four areas (Haman, Choongju, Uljin, and Pohang) of the Republic of Korea. Baseline information on general lifestyle, physical activity, diet, reproductive factors, and others were collected by direct interview. Blood (plasma, or serum buffy coat, packed erythrocytes) and urine samples were also obtained. Based on data linkage with the national cancer registry, death-certificate system, and health-insurance databases, study participants were regularly followed-up. The final updates were done to identify all deaths that occurred until the end of 2014 and cancers that occurred until the end of 2013. This study is still ongoing.	Asian Pac J Cancer Prev. 2002;3(1):85-92.rs
Seoul Male Cohort Study<br>(Seoul Male)	• This study was initiated in 1992, with an enrollment of a total of 29,918 men aged 40-59. Baseline information was collected by self-administered questionnaires from 14,533 participants. Death certificates from the National Statistics Office were used to ascertain vital status. Study participants were followed-up from January 1, 1993 to December 31, 2008.	J Prev Med Public Health. 2012;45(1):14-20.
Singapore Chinese Health Study<br>(SCHS)	• This study aims to investigate the role of diet and genetic factors in cancer etiology. Between 1993 and 1999, a total of 63,257 men and women, aged 45-74, were recruited for this cohort. At the recruitment, participants were interviewed using staff-administered questionnaires including a validated Food Frequency Questionnaire. Biologic samples were also obtained from consenting cohort members. Study participants were regularly followed-up for cancer incidence and mortality via record linkages to local cancer and vital status registries.	Nutr Cancer. 2001;39(2):187-95. doi: 10.1207/S15327914nc392_5
Community-Based Cancer Screening Project<br>(CBCSP)	• From January 1991 to December 1992, a total of 23,820 individuals, aged 30-65, were recruited from seven townships in Taiwan. Using structured questionnaires, baseline and follow-up information were administered by trained staff. Participants were followed-up for cancer incidence and death through health examination, medical record review, and data linkage to the national cancer registry and death certification systems.	JAMA. 2006; 295(1):65-73. doi: 10.1001/jama.295.1.65
CardioVascular Disease risk FACtor Two-township Study<br>(CVDFACTS)	• Between 1990-1993, a total of 5,160 adults were recruited from Chu-Don, a Hakka community in northwest Taiwan and from Pu-Tze, a Fukien community in southern Taiwan. In each of the two communities, five villages with >1,000 people or with a population density >200 per square kilometer were randomly selected for this study. Follow-up is conducted every three years using death certificates provided by the Department of Health in Taiwan.	Stroke. 2009;40(5):1578-84. doi: 10.1161/STROKEAHA.108.540492
Mumbai Cohort Study<br>(Mumbai)	• This study recruited participants in two phases, 1991-1994 and 1994-1996. Participants were residents of Mumbai and ≥35 years of age. About 150,000 subjects were interviewed at baseline. To ascertain participants' vital status, an active house-to-house follow-up was done after an average of 5.5 years. Data on cause of death was obtained from the local death registry. Cancer incidence was ascertained by data linkage to cancer registry databases. This study is still ongoing.	Int J Epidemiol. 2008;37(3):524-35. doi: 10.1093/ije/dyn001.

© 2019 Yang JJ et al. *JAMA Network Open.*

eTable 2. Baseline Characteristics Across Birth Cohorts

Birth Cohorts	No. of Participants [a]	No. of Men	No. of Women	Follow-up Years	Age at Baseline [b]	No. of Deaths			No. of Participants [a] by Country/Region				
						All causes	Lung Cancer		China (Urban)	China (Rural)	Japan	Korea/Singapore/Taiwan	India
**<1910**	18,912	8,967	9,945	9.6	76.2	16,610	480		932	683	16,559	-	696
**1910-1919**	51,667	23,515	28,152	10.3	72.7	24,897	993		5,480	4,303	37,646	711	3,569
**1920-1929**	174,155	82,729	91,426	11.6	64.7	42,876	2,894		24,438	10,841	111,211	13,068	14,597
**1930-1939**	297,698	146,264	151,434	12.5	56.9	38,946	3,248		61,594	16,187	154,859	34,554	30,504
**1940-1949**	298,406	149,475	148,931	12.1	48.0	16,745	1,197		61,577	21,572	126,326	42,304	46,627
**≥1950**	161,420	79,337	82,083	10.7	42.5	4,292	346		68,522	2,883	27,886	14,904	47,225
**TOTAL**	1,002,258	490,287	511,971	11.7	54.5	144,366	9,158		222,543	56,469	474,487	105,541	143,218

[a] Including only participants eligible for the current analysis
[b] Mean age at the enrollment of baseline (smoking) survey

© 2019 Yang JJ et al. *JAMA Network Open.*

eTable 3. Mean Age[a] at Quitting Tobacco Smoking Among Former Smokers by Birth Cohorts and Study Populations: Asian Male Populations

		Birth Cohorts						
**Populations**	**Baseline Age group**	**<1910**	**1910-1919**	**1920-1929**	**1930-1939**	**1940-1949**	**≥1950**	***P*** [b]
China – Urban area	<50 yrs.	-[c]	-[c]	-[c]	43.2 (41.2-45.1)	39.7 (39.0-40.4)	40.0 (39.7-40.4)	<0.01
	50-59 yrs.	-[c]	-[c]	49.9 (48.3-51.4)	47.5 (46.9-48.0)	49.2 (48.7-49.7)	44.6 (44.1-45.1)	<0.01
	60-69 yrs.	-[c]	-[c]	55.0 (54.5-55.6)	56.9 (56.3-57.4)	53.7 (53.0-54.5)	-[c]	<0.01
	≥70 yrs.	69.5 (63.8-75.1)	65.0 (63.6-66.4)	60.9 (59.5-62.2)	60.3 (59.6-60.9)	-[c]	-[c]	<0.01
China – Rural area	<50 yrs.	-[c]	-[c]	-[c]	-[c]	41.5 (40.3-42.8)	36.4 (31.9-40.8)	0.01
	50-59 yrs.	-[c]	-[c]	-[c]	50.3 (49.1-51.6)	43.3 (41.0-45.5)	-[c]	<0.01
	60-69 yrs.	-[c]	-[c]	57.8 (56.4-59.1)	54.2 (51.4-57.1)	-[c]	-[c]	0.07
	≥70 yrs.	72.2 (59.1-85.3)	65.1 (62.8-67.4)	62.4 (58.2-66.6)	-[c]	-[c]	-[c]	0.09
	<50 yrs.	-[c]	-[c]	35.8 (34.9-36.6)	38.0 (37.6-38.3)	35.5 (35.4-35.7)	33.0 (32.6-33.4)	<0.01
Japan	50-59 yrs.	-[c]	-[c]	47.1 (46.6-47.6)	43.9 (43.7-44.1)	42.1 (41.7-42.6)	45.0 (43.4-46.7)	<0.01
	60-69 yrs.	-[c]	55.3 (54.5-56.1)	52.6 (52.3-52.9)	49.9 (49.5-50.3)	51.5 (50.6-52.3)	50.4 (48.2-52.6)	<0.01
	≥70 yrs.	63.5 (62.4-64.6)	60.1 (59.5-60.8)	58.0 (57.2-58.8)	-[c]	-[c]	-[c]	<0.01
Korea/Singapore/Taiwan	<50 yrs.	-[c]	-[c]	-[c]	-[c]	37.3 (37.0-37.7)	34.7 (34.1-35.2)	<0.01
	50-59 yrs.	-[c]	-[c]	-[c]	45.5 (45.0-45.9)	42.1 (41.6-42.5)	38.7 (34.7-42.7)	<0.01
	60-69 yrs.	-[c]	-[c]	52.4 (51.6-53.2)	49.5 (48.9-50.2)	51.9 (48.4-55.5)		<0.01
	≥70 yrs.	-[c]	60.1 (56.0-64.1)	55.1 (54.1-56.2)	57.9 (54.3-61.6)			0.02
India	<50 yrs.	-[c]	-[c]	-[c]	-[c]	37.6 (36.9-38.3)	33.9 (33.2-34.5)	<0.01
	50-59 yrs.	-[c]	-[c]	-[c]	45.4 (44.6-46.2)	42.2 (41.7-42.8)	-[c]	<0.01
	60-69 yrs.	-[c]	-[c]	51.8 (50.9-52.7)	50.6 (50.1-51.2)	-[c]	-[c]	0.03
	≥70 yrs.	67.6 (64.0-71.2)	59.2 (57.7-60.7)	57.0 (56.1-57.8)	-[c]	-[c]	-[c]	<0.01

[a] Mean (95% confidence interval) age at quitting smoking among former-smokers

[b] Estimated by using Analysis of variance (ANOVA) test

[c] No data available

© 2019 Yang JJ et al. *JAMA Network Open.*

eTable 4. Risk of Death Associated With Tobacco Smoking by Birth Cohorts and Attained Age in Asian Male Populations: Current vs Never Smokers

		Hazard Ratios (95% Confidence Intervals)					
		<1920		1920-1929		≥1930	
**Population**	**Attained Age group [a]**	**No. of Death**	**HR (95% CI) [b]**	**No. of Death**	**HR (95% CI) [b]**	**No. of Death**	**HR (95% CI) [b]**
**Death from All Causes**							
China – Urban area	50-59 yrs.	0	NA[c]	12	NA[c]	1,668	1.23 (1.10-1.38)
	60-69 yrs.	0	NA[c]	1,294	0.98 (0.86-1.12)	2,403	1.35 (1.23-1.47)
	70-79 yrs.	426	1.04 (0.85-1.28)	2,565	1.36 (1.25-1.47)	1,587	1.60 (1.45-1.78)
Japan	50-59 yrs.	203	1.17 (0.72-1.90)	238	0.98 (0.51-1.86)	3,641	1.33 (1.22-1.45)
	60-69 yrs.	1,110	1.14 (0.92-1.40)	2,189	1.17 (1.02-1.33)	7,039	1.60 (1.43-1.78)
	70-79 yrs.	4,214	1.08 (0.96-1.21)	6,422	1.39 (1.17-1.64)	4,637	1.84 (1.71-1.99)
Korea/Singapore/Taiwan	50-59 yrs.	0	NA[c]	0	NA[c]	1,095	1.40 (1.23-1.61)
	60-69 yrs.	0	NA[c]	245	1.13 (0.66-1.94)	2,454	1.43 (1.23-1.67)
	70-79 yrs.	50	0.75 (0.26-2.21)	1,436	1.16 (0.92-1.45)	1,106	2.00 (1.74-2.29)
India	50-59 yrs.	0	NA[c]	0	NA[c]	1,703	1.40 (1.27-1.55)
	60-69 yrs.	0	NA[c]	540	1.10 (0.91-1.32)	1,634	1.28 (1.15-1.42)
	70-79 yrs.	276	1.08 (0.81-1.44)	1,401	1.16 (1.02-1.31)	53	1.53 (0.85-2.74)
**Death from Lung Cancer [d]**							
China – Urban area	50-59 yrs.	0	NA[c]	2	NA[c]	235	3.16 (1.97-5.05)
	60-69 yrs.	0	NA[c]	168	3.30 (1.65-6.59)	363	4.36 (2.22-8.55)
	70-79 yrs.	27	3.78 (1.62-8.85)	280	3.97 (2.90-5.43)	248	4.91 (3.52-6.83)
Japan	50-59 yrs.	7	NA[c]	13	NA[c]	243	3.34 (1.92-5.81)
	60-69 yrs.	68	1.94 (0.60-6.28)	249	2.85 (1.42-5.71)	683	4.48 (3.26-6.15)
	70-79 yrs.	337	5.45 (3.01-9.87)	637	3.66 (2.34-5.74)	516	6.05 (2.68-13.6)
Korea/Singapore/Taiwan	50-59 yrs.	0	NA[c]	0	NA[c]	115	2.54 (0.75-8.59)
	60-69 yrs.	0	NA[c]	29	2.97 (1.01-8.75)	316	3.25 (1.80-5.85)
	70-79 yrs.	6	NA[c]	187	3.32 (0.76-14.5)	157	5.08 (2.32-11.1)

HR = hazard ratio; 95% CI = 95% confidence interval; NA = not available;

[a] Age at date of death, end of follow-up, or follow-up loss

[b] Adjusted for age, education, marital status, rural/urban residence, and body mass index; and stratified by five-year groups of birth year and enrollment year

[c] Not estimated due to small sample size (<20 events)

[d] The number of deaths due to lung cancer were less than 20 for any of the birth cohorts included in the analysis for Indians, and thus no HR was estimated

© 2019 Yang JJ et al. *JAMA Network Open.*

eTable 5. Risk of Death Associated With Tobacco Smoking History by Birth Cohorts in Asian Male Populations

	Hazard Ratios (95% Confidence Intervals)					
	<1920		1920-1929		≥1930	
**Smoking Characteristics**	**No. of Death**	**HR (95% CI) [a]**	**No. of Death**	**HR (95% CI) [a]**	**No. of Death**	**HR (95% CI) [a]**
**Death from All Causes**						
Age at starting smoking (year)						
≥ 30	840	1.04 (0.96-1.13)	1,485	1.18 (1.05-1.33)	2,304	1.26 (1.16-1.37)
25-29	640	1.18 (1.08-1.29)	1,865	1.32 (1.20-1.44)	3,178	1.45 (1.35-1.55)
20-24	2,546	1.20 (1.09-1.33)	5,520	1.34 (1.28-1.40)	9,981	1.55 (1.44-1.66)
< 20	975	1.30 (1.12-1.50)	3,626	1.50 (1.35-1.66)	6,872	1.75 (1.61-1.91)
P for trend		<0.01		<0.01		<0.01
No. of cigarettes smoked per day						
0-9	2,199	1.20 (1.09-1.31)	2,754	1.25 (1.17-1.33)	3,840	1.35 (1.27-1.43)
10-19	5,828	1.17 (1.06-1.29)	4,849	1.38 (1.26-1.50)	6,494	1.50 (1.40-1.61)
20-29	3,009	1.24 (1.14-1.35)	4,985	1.41 (1.29-1.55)	9,302	1.63 (1.51-1.75)
≥ 30	1,030	1.28 (1.15-1.43)	2,044	1.42 (1.27-1.58)	5,257	1.79 (1.65-1.93)
P for trend		<0.01		<0.01		<0.01
**Death from Lung Cancer**						
Age at starting smoking (year)						
≥ 30	30	2.01 (1.00-4.05)	108	2.32 (1.63-3.32)	196	2.63 (1.94-3.55)
25-29	31	3.25 (1.87-5.64)	214	3.60 (2.67-4.86)	357	3.79 (2.95-4.87)
20-24	190	3.90 (2.66-5.73)	634	3.92 (3.00-5.12)	1,130	4.64 (3.61-5.95)
< 20	60	3.36 (1.67-6.77)	485	4.90 (3.76-6.39)	894	5.78 (4.55-7.35)
P for trend		<0.01		<0.01		<0.01
No. of cigarettes smoked per day						
0-9	65	1.93 (1.34-2.77)	166	2.27 (1.63-3.16)	189	1.90 (1.47-2.45)
10-19	339	2.96 (2.22-3.95)	545	3.83 (2.97-4.94)	652	3.60 (2.96-4.38)
20-29	242	4.11 (3.08-5.49)	658	4.69 (3.51-6.25)	1,194	4.96 (3.93-6.25)
≥ 30	102	6.06 (4.21-8.72)	307	5.65 (4.21-7.60)	801	7.83 (6.19-9.91)
P for trend		<0.01		<0.01		<0.01

HR = hazard ratio; 95% CI = 95% confidence interval; Never-smokers were used as a reference group for each analysis

[a] Adjusted for age, education, marital status, rural/urban residence, and body mass index; and stratified by five-year groups of birth year and enrollment year

© 2019 Yang JJ et al. *JAMA Network Open.*

eTable 6. Risk of Death Associated With Age at Quitting Smoking by Birth Cohorts in Asian Male Populations

	Hazard Ratios (95% Confidence Intervals)					
	<1920		1920-1929		≥1930	
**Age at quitting smoking (year)**	**No. of Death**	**HR (95% CI) [a]**	**No. of Death**	**HR (95% CI) [a]**	**No. of Death**	**HR (95% CI) [a]**
			**Death from All Causes**			
≥ 60	1,473	1.29 (1.21-1.39)	1,543	1.38 (1.23-1.54)	598	1.44 (1.21-1.72)
50-59	601	1.10 (0.92-1.31)	1,265	1.22 (1.08-1.38)	1,627	1.56 (1.45-1.68)
40-49	320	1.03 (0.87-1.22)	535	1.06 (0.91-1.24)	1,347	1.21 (1.12-1.32)
< 40	217	1.06 (0.92-1.22)	351	1.00 (0.89-1.12)	910	1.03 (0.92-1.16)
Never-Smoker	5,212	1 (reference)	6,213	1 (reference)	9,646	1 (reference)
P for trend		<0.01		<0.01		<0.01
			**Death from Lung Cancer**			
≥ 60	76	3.84 (2.60-5.69)	147	3.24 (2.46-4.27)	77	4.04 (2.82-5.78)
50-59	26	3.49 (1.61-7.55)	82	2.13 (1.59-2.86)	129	3.20 (2.48-4.13)
40-49	8	NA[b]	21	1.06 (0.64-1.77)	58	1.62 (1.12-2.34)
< 40	6	NA[b]	8	NA[b]	40	1.48 (1.02-2.14)
Never-Smoker	39	1 (reference)	197	1 (reference)	346	1 (reference)
P for trend		<0.01		<0.01		<0.01

HR = hazard ratio; 95% CI = 95% confidence interval; NA= not available;

[a] Adjusted for age, education, marital status, rural/urban residence, and body mass index; and stratified by five-year groups of birth year and enrollment year

[b] Not estimated due to small sample size (<20 events)

© 2019 Yang JJ et al. *JAMA Network Open.*

eTable 7. Risk of Death Associated With Tobacco Smoking History by Birth Cohorts in Asian Female Populations

	Hazard Ratios (95% Confidence Intervals)							
	<1920			1920-1929			≥1930	
**Smoking Characteristics**	**No. of Death**	**HR (95% CI) [a]**		**No. of Death**	**HR (95% CI) [a]**		**No. of Death**	**HR (95% CI) [a]**
**Death from All Causes**								
Age at starting smoking (year)								
≥ 30	554	1.15 (1.04-1.28)		585	1.32 (1.21-1.44)		797	1.49 (1.33-1.67)
20-29	284	1.29 (1.12-1.49)		474	1.61 (1.46-1.77)		689	1.72 (1.48-2.00)
< 20	205	1.32 (1.14-1.52)		402	1.88 (1.51-2.35)		420	1.93 (1.64-2.27)
P for trend		<0.01			<0.01			<0.01
No. of cigarettes smoked per day								
0-9	1,286	1.27 (1.20-1.35)		845	1.32 (1.23-1.42)		809	1.43 (1.33-1.53)
10-19	886	1.30 (1.18-1.43)		752	1.64 (1.52-1.77)		812	1.71 (1.52-1.92)
≥ 20	370	1.22 (1.10-1.35)		400	1.66 (1.46-1.88)		608	1.91 (1.65-2.21)
P for trend		<0.01			<0.01			<0.01
**Death from Lung Cancer**								
Age at starting smoking (year)								
≥ 30	18	1.89 (1.11-3.24)		35	2.34 (1.63-3.35)		69	2.41 (1.88-3.10)
20-29	16	3.86 (2.01-7.42)		52	4.66 (3.38-6.43)		80	4.07 (3.21-5.16)
< 20	13	3.94 (2.09-7.41)		66	7.40 (4.40-12.5)		64	4.98 (3.34-7.44)
P for trend		<0.01			<0.01			<0.01
No. of cigarettes smoked per day								
0-9	43	2.74 (1.62-4.63)		67	2.96 (1.98-4.44)		75	2.43 (1.90-3.11)
10-19	44	3.45 (2.47-4.82)		89	5.79 (4.50-7.44)		85	3.47 (2.76-4.36)
≥ 20	24	4.35 (2.43-7.78)		40	5.43 (3.83-7.71)		83	5.61 (4.16-7.58)
P for trend		<0.01			<0.01			<0.01

HR = hazard ratio; 95% CI = 95% confidence interval; Never-smokers were used as a reference group for each analysis

[a] Adjusted for age, education, marital status, rural/urban residence, and body mass index; and stratified by five-year groups of birth year and enrollment year

© 2019 Yang JJ et al. *JAMA Network Open.*

eTable 8. Risk of Death Associated With Tobacco Smoking by Birth Cohorts in Asian Populations: Sensitivity Analysis Not Adjusted for Body Mass Index

		**Hazard Ratios (95% Confidence Intervals)**					
		**<1920**		**1920-1929**		**≥1930**	
	**No. of Participants**	**No. of Death**	**HR (95% CI) [a]**	**No. of Death**	**HR (95% CI) [a]**	**No. of Death**	**HR (95% CI) [a]**
**Death from All Causes**							
**Male**							
Never-Smoker	169,444	5,212	1 (reference)	6,213	1 (reference)	9,646	1 (reference)
Ever-Smoker	320,843	15,986	1.24 (1.16-1.31)	19,786	1.39 (1.30-1.47)	28,884	1.60 (1.52-1.70)
Current Smoker	249,241	11,587	1.29 (1.19-1.39)	14,299	1.49 (1.36-1.62)	23,468	1.72 (1.59-1.85)
**Female**							
Never-Smoker	472,081	17,151	1 (reference)	14,532	1 (reference)	18,946	1 (reference)
Ever-Smoker	39,890	3,158	1.28 (1.23-1.33)	2,345	1.51 (1.44-1.58)	2,507	1.69 (1.57-1.82)
Current Smoker	33,719	2,472	1.31 (1.24-1.37)	1,956	1.54 (1.46-1.63)	2,173	1.74 (1.60-1.89)
**Death from Lung Cancer**							
**Male**							
Never-Smoker	169,444	93	1 (reference)	197	1 (reference)	346	1 (ref.)
Ever-Smoker	320,843	967	3.01 (2.25-4.04)	2,043	3.83 (3.01-4.86)	3,133	4.20 (3.33-5.28)
Current Smoker	249,241	765	3.44 (2.30-5.15)	1,705	4.78 (3.62-6.30)	2,797	4.93 (3.81-6.37)
**Female**							
Never-Smoker	472,081	289	1 (reference)	437	1 (reference)	1,059	1 (reference)
Ever-Smoker	39,890	129	2.91 (2.22-3.81)	217	3.88 (3.01-5.00)	253	3.26 (2.80-3.79)
Current Smoker	33,719	104	2.97 (2.36-3.75)	186	4.20 (3.20-5.51)	233	3.58 (2.99-4.28)

HR = hazard ratio; 95% CI = 95% confidence interval; NA= not available;

[a] Adjusted for age, education, marital status, and rural/urban residence; and stratified by five-year groups of birth year and enrollment year

© 2019 Yang JJ et al. *JAMA Network Open.*

eTable 9. Risk of Death Associated With Tobacco Smoking by Birth Cohorts in Asian Populations: Sensitivity Analysis Further Adjusted for Physical Activity Status

			Hazard Ratios (95% Confidence Intervals)							
			<1920			1920-1929			≥1930	
	**No. of Participants**		**No. of Death**	**HR (95% CI) [a]**		**No. of Death**	**HR (95% CI) [a]**		**No. of Death**	**HR (95% CI) [a]**
**Death from All Causes**										
**Male**										
Never-Smoker	169,444		5,212	1 (reference)		6,213	1 (reference)		9,646	1 (reference)
Ever-Smoker	320,843		15,986	1.22 (1.15-1.30)		19,786	1.37 (1.29-1.46)		28,884	1.59 (1.50-1.68)
Current Smoker	249,241		11,587	1.26 (1.21-1.31)		14,299	1.49 (1.42-1.56)		23,468	1.66 (1.54-1.79)
**Female**										
Never-Smoker	472,081		17,151	1 (reference)		14,532	1 (reference)		18,946	1 (reference)
Ever-Smoker	39,890		3,158	1.26 (1.21-1.31)		2,345	1.48 (1.42-1.55)		2,507	1.65 (1.53-1.78)
Current Smoker	33,719		2,472	1.29 (1.23-1.35)		1,956	1.52 (1.43-1.61)		2,173	1.71 (1.57-1.86)
**Death from Lung Cancer**										
**All population**										
Never-Smoker	169,444		93	1 (reference)		197	1 (reference)		346	1 (ref.)
Ever-Smoker	320,843		967	3.00 (2.24-4.02)		2,043	3.77 (2.94-4.83)		3,133	4.09 (3.25-5.14)
Current Smoker	249,241		765	3.35 (2.23-5.03)		1,705	4.71 (3.55-6.26)		2,797	4.79 (3.70-6.18)
**Female**										
Never-Smoker	472,081		289	1 (reference)		437	1 (reference)		1,059	1 (reference)
Ever-Smoker	39,890		129	2.86 (2.20-3.72)		217	3.82 (3.00-4.86)		253	3.21 (2.77-3.72)
Current Smoker	33,719		104	2.92 (2.31-3.69)		186	4.14 (3.20-5.35)		233	3.53 (2.99-4.17)

HR = hazard ratio; 95% CI = 95% confidence interval; NA= not available;
[a] Adjusted for age, education, marital status, rural/urban residence, body mass index, and leisure time physical activity; and stratified by five-year groups of birth year and enrollment year

© 2019 Yang JJ et al. *JAMA Network Open.*

eTable 10. Risk of Death Associated With Tobacco Smoking by Birth Cohorts in Asian Populations: Sensitivity Analysis Using the Multiple Imputation Method

		Hazard Ratios (95% Confidence Intervals)					
		<1920		1920-1929		≥1930	
	No. of Participants	No. of Death	HR (95% CI) [a]	No. of Death	HR (95% CI) [a]	No. of Death	HR (95% CI) [a]
		Death from All Causes					
**Male**							
Never-Smoker	169,444	5,212	1 (reference)	6,213	1 (reference)	9,646	1 (reference)
Ever-Smoker	320,843	15,986	1.22 (1.14-1.30)	19,786	1.37 (1.29-1.46)	28,884	1.59 (1.50-1.68)
Current Smoker	249,241	11,587	1.27 (1.17-1.37)	14,299	1.47 (1.35-1.61)	23,468	1.70 (1.57-1.85)
**Female**							
Never-Smoker	472,081	17,151	1 (reference)	14,532	1 (reference)	18,946	1 (reference)
Ever-Smoker	39,890	3,158	1.26 (1.22-1.31)	2,345	1.49 (1.43-1.56)	2,507	1.66 (1.54-1.79)
Current Smoker	33,719	2,472	1.29 (1.23-1.35)	1,956	1.52 (1.43-1.62)	2,173	1.71 (1.57-1.86)
		Death from Lung Cancer					
**All population**							
Never-Smoker	169,444	93	1 (reference)	197	1 (reference)	346	1 (ref.)
Ever-Smoker	320,843	967	3.03 (2.26-4.06)	2,043	3.77 (2.94-4.83)	3,133	4.09 (3.25-5.15)
Current Smoker	249,241	765	3.40 (2.26-5.11)	1,705	4.73 (3.55-6.30)	2,797	4.79 (3.71-6.19)
**Female**							
Never-Smoker	472,081	289	1 (reference)	437	1 (reference)	1,059	1 (reference)
Ever-Smoker	39,890	129	2.86 (2.21-3.70)	217	3.83 (3.02-4.85)	253	3.22 (2.78-3.73)
Current Smoker	33,719	104	2.91 (2.30-3.67)	186	4.15 (3.22-5.34)	233	3.55 (3.01-4.17)

HR = hazard ratio; 95% CI = 95% confidence interval; NA= not available;

[a] Adjusted for age, education, marital status, rural/urban residence, and body mass index; and stratified by five-year groups of birth year and enrollment year

© 2019 Yang JJ et al. *JAMA Network Open.*
